# Reirradiation based on diffusion-weighted magnetic resonance imaging-guided dose- painting for locally advanced recurrent nasopharyngeal carcinoma: a phase 2 randomized controlled trial

**DOI:** 10.1186/s12885-025-13969-5

**Published:** 2025-04-07

**Authors:** Chao Tan, Yuyi Li, Xiaoyu Chen, Weichang Zhu, Cuihong Jiang, Lili He, Shuai Xiao, Changgen Fan, Xu Ye, Qi Zhao, Wenqiong Wu, Yanxian Li, Yanfang Qiu, Kailin Chen, Shulu Hu, Feng Liu, Hui Wang

**Affiliations:** 1 Department of Radiation Oncology, The Affiliated Cancer Hospital of Xiangya School of Medicine, Central South University/Hunan Cancer Hospital, Changsha, Hunan China; 2 Graduate Collaborative Training Base of Hunan Cancer Hospital, Hengyang Medical School, University of South China, Hengyang, Hunan China; 3 Hunan Key Laboratory of Translational Radiation Oncology, The Affiliated Cancer Hospital of Xiangya School of Medicine, Hunan Cancer Hospital, Central South University, Changsha, Hunan China

**Keywords:** Recurrent NPC, Reirradiation (ReRT), DWI (diffusion weighted imaging), IMRT (intensity modulated radiation therapy), Toxicicity

## Abstract

**Introduction:**

The effect of diffusion-weighted magnetic resonance imaging (DWI)-guided dose-painting intensity-modulated radiation therapy (DP-IMRT) on locally advanced recurrent nasopharyngeal carcinoma (NPC) remains unclear. This study aimed to compare the outcomes and toxicities of DWI-guided DP-IMRT in patients with locally recurrent NPC.

**Methods:**

In this prospective trial, 150 patients with locally advanced recurrent NPC were randomly assigned (1:1) to receive reirradiation with DWI-guided DP-IMRT (DWI group, *n* = 75) or conventional MRI-based IMRT (MRI group, *n* = 75). In the DWI group, DWI-guided gross tumor volume received escalation to 65.4 Gy/30 fx in 2.18 Gy per fraction, while in the MRI group, the planning target volume was irradiated at 60 Gy/30fx in 2.0 Gy per fraction. The trial was registered at Chictr.org.cn (ChiCTR2100052340) on October 24, 2021. Survival rates were compared, and multivariate analyses were conducted.

**Results:**

The median follow-up duration was 16 months. Compared with the MRI group, patients in the DWI group had better 18-month progression-free survival (PFS) 75.1% vs. 53.6%; *P* = 0.006), local recurrence-free survival (LRFS) (83.4% vs. 61.8%; *P* = 0.010), and locoregional recurrence-free survival (73.1% vs. 64.9%; *P* = 0.025). Grade 3–4 toxicities between the two groups showed no significant difference. Multivariate analysis revealed that DWI-guided DP-IMRT was an independent prognostic factor for PFS and LRFS.

**Conclusion:**

Compared with conventional MRI-based IMRT, DWI-guided DP-IMRT improved PFS in patients with recurrent NPC without increasing acute and late toxic effects.

**Supplementary Information:**

The online version contains supplementary material available at 10.1186/s12885-025-13969-5.

## Introduction

Nasopharyngeal carcinoma (NPC) is one of the most common head and neck tumors endemic to Southeast Asia [1, 2]. Radiotherapy or chemoradiotherapy is recommended as the primary treatment option for NPC [3]. Recurrence is the primary cause of treatment failure; the incidence of local recurrence after primary radical radiotherapy in patients with NPC is 10–20% [4–6]. Several studies have shown that endoscopic surgery is the recommended treatment for resectable locally recurrent NPC [7–9]; however, reirradiation is the only effective salvage option with curative intent in patients with unresectable locally advanced lesions [10]. Despite recent advances, tumor control remains a challenge in the treatment of locally recurrent NPC. Boosting the radiation dose to the gross tumor volume (GTV) has demonstrated potential for enhancing local control (LC).

The standard method for target volume delineation is based on computed tomography (CT) and conventional magnetic resonance imaging (MRI) [11]. One useful method to improve tumor efficacy by increasing the radiation dose is through dose painting (DP), which is achieved using functional imaging. Diffusion-weighted magnetic resonance imaging (DWI) has been proposed as a candidate method for diagnosing DP. Several studies indicated that pretreatment DWI was significantly associated with treatment efficacy in NPC [12, 13]. The apparent diffusion coefficient (ADC) targets of tumor lesions have been shown to function as markers for predicting the response to chemoradiation in NPC and other head and neck squamous cell carcinomas (HNSCCs) [14]. A retrospective study [12] demonstrated that DWI-guided dose escalation is a practical method capable of enhancing local control in patients with locoregionally advanced nasopharyngeal carcinoma (LA-NPC) while being tolerable in terms of treatment-related complications. Our previous studies have shown that DWI-guided DP-intensity-modulated radiation therapy (IMRT) is a prognostic factor for disease-free survival (DFS) in patients with LA-NPC.

We designed this trial for locally recurrent NPC treated with radiotherapy to compare the outcomes and toxicities of DWI-guided DP-IMRT and conventional MRI-based IMRT. We hypothesized that DWI-guided DP-IMRT would increase the efficacy, but not the incidence, of radiation-induced toxicity.

## Materials and methods

### Study design

**Fig. 1:**
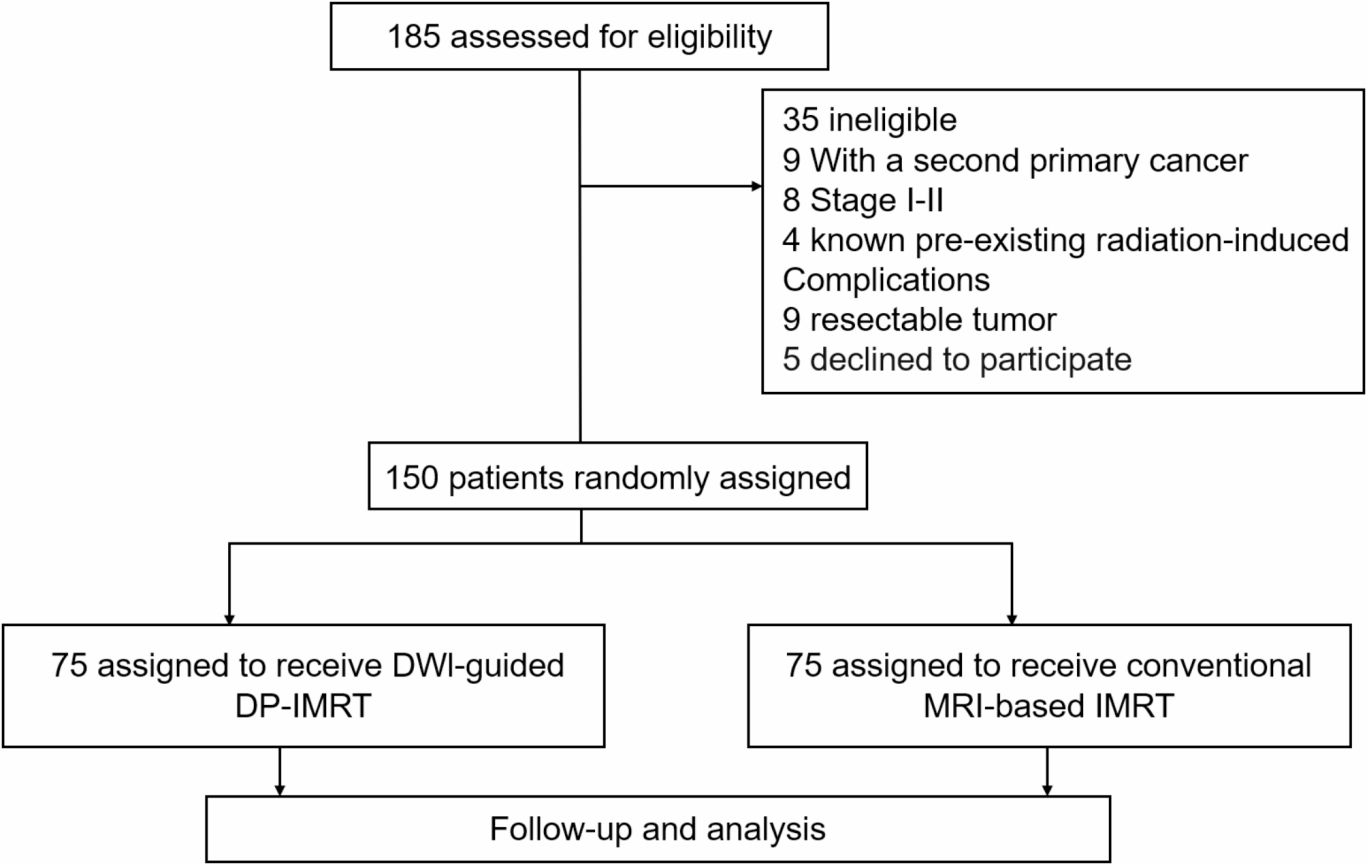
Trial profile

This phase 2 randomized clinical trial was conducted at the Hunan Cancer Hospital and The Affiliated Cancer Hospital of Xiangya School of Medicine, Central South University. We used the Excel Rand function to produce random numbers for registration and randomization. Patients were randomly assigned (1:1) to the DWI group (DWI-guided DP-IMRT) or the MRI group (conventional MRI-based IMRT) (Fig. 1). This study was conducted in accordance with the Declaration of Helsinki and approved by the Ethics Committee of Hunan Cancer Hospital. The trial was registered at Chictr.org.cn (ChiCTR2100052340) on October 24, 2021.

### Participants

All patients underwent a pretreatment evaluation, including a complete history, systematic physical examination, nasopharyngeal endoscopy, and MRI. Eligibility criteria included undifferentiated or differentiated stage rT3-4N0-3M0 (8th International Union Against Cancer); recurrent NPC confirmed by pathology or histology; age between 18 and 70 years; previous radical radiotherapy before recurrence; interval between recurrence and prior radiotherapy > 1 year; Karnofsky performance status score of ≥ 70; normal function of major organs as evidenced by routine blood and biochemical tests meeting standards, and written informed consent. The exclusion criteria included evidence of distant metastasis, history of previous or synchronous malignant tumors, presence of active infection or unstable cardiac disease, and pregnancy or lactation.

### Imaging

All patients underwent contrast-enhanced MRI of the head and neck using a 1.5-Tesla scanner (Magnetom Sonata; Siemens, Erlangen, Germany). MRI included T1- and T2-weighted imaging, T1-contrast-enhanced sequences, and DWI. An ADC map was constructed using a scanner. CT simulation images were fused with T1-weighted MR images in both groups. In the DWI group, DWI images (b-values = 0 and 1000 s/mm^2^) were fused to the CT simulation images.

We used deformable registration of the DWI image (b = 0 s/mm^2^ image) to T1-weighted MRI to correct the distortion caused by sensitive artifacts. Deformable registration was performed using a six-step multiresolution strategy based on mutual information, gradient-based optimizer, and global smoothness penalty [15].

### Radiotherapy

**Fig. 2:**
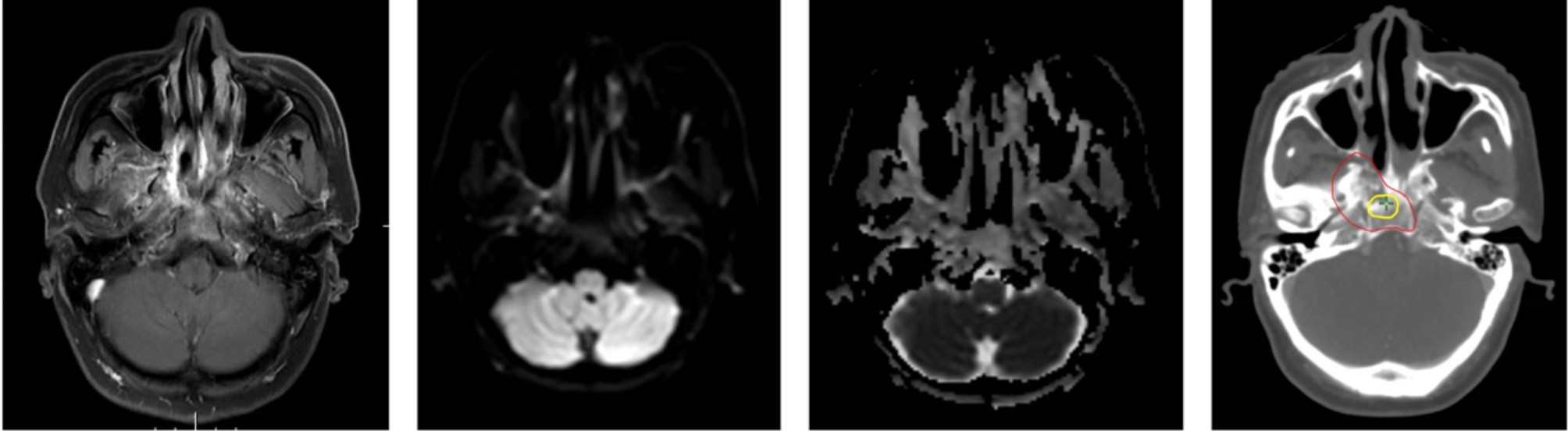
Example of target mapping of IMRT plan for a patient with rT3N2M0 nasopharynx carcinoma by diffusion-weighted magnetic resonance imaging guided dose mapping before induction chemotherapy. (**A**) Contrast-enhanced T1-weighted sequence. (**B**) DWI sequence. (**C**) ADC map. (**D**) Transverse slice of target volumes. Targets: high-dose gross target volume (GTV-dwi) (yellow) and gross tumor volume (GTV) (red) were delineated at apparent diffusion coefficient map fused with computed tomography simulation image

All patients underwent IMRT using a 6 MV photon beam and a linear accelerator. The GTV was defined based on the fiberoptic nasopharyngoscopy, planning CT, and MRI data (Fig. 2). The clinical target volume (CTV) was defined as the GTV and subclinical lesions with a 5 mm margin. CTV included the entire nasopharynx and the lymph node-positive involved regions. The planning target volume (PTV) was defined as the safety boundary (3 mm) outside the CTV.

In DWI, the ADCmean is defined as the mean ADC value within the GTV on an ADC map [16]; however, large variations have been reported in ADC values in the head and neck (HN) area, ranging from 0.4 × 10^3^ mm^2^/s to 1.4 × 10^3^ mm^2^/s [17]. Moreover, a low ADC indicates that tumor presence is associated with worse outcomes [18, 19]. Thus, areas within the GTV with an ADC below the mean ADC were segmented as GTV-dwi according to the MRI before treatment. GTV contours were manually delineated by two radiation oncologists specializing in the HN region.

The prescribed GTV-dwi and PTV doses for patients in the DWI group were 65.4 Gy, using 2.18 Gy per fraction and 60 Gy/30 fx, respectively. The prescribed PTV for patients in the MRI group was 60 Gy/30 fx. Both groups received the same duration of radiotherapy. The dose constraints of the neurological organs at risk (OARs) refer to international recommendations on reirradiation by IMRT for locally recurrent NPC [20] and previous studies [21, 22]. The lifetime cumulative dose should not exceed 130% of the single-dose maximum tolerated dose. Dose limitation to the brain stem and spinal cord were 40 Gy and 30 Gy to Dmax, respectively. The maximum dose for other OARs were based on TD5/5, considering previous irradiated dose with 70% recovery.

### Chemotherapy

The induction chemotherapy (IC) regimen was GP (gemcitabine at 1 g/m^2^ on days 1 and 8, and cisplatin at 80 mg/m^2^ on day 1). All patients were scheduled to receive three cycles except for those who refused to undergo chemotherapy or had severe adverse events.

### Follow-up

The follow-up duration began on the date of randomization and ended on the last follow-up date (December 1, 2023) or until death. All acute and late adverse events were evaluated based on the Common Terminology Criteria for Adverse Events (version 5.0) and Radiation Therapy Oncology Group (RTOG) acute and late radiation morbidity scoring criteria [23], respectively. And late toxicities that occurred 3 months after completion of radiotherapy until the latest follow-up were graded. All patients underwent evaluation every 3 months for the first 3 years and every 6 months in the subsequent years. Salvage treatments, such as surgery and chemotherapy, were implemented in patients with confirmed tumor progression.

### Statistical analysis

The primary endpoints were progression-free survival (PFS) and local recurrence-free survival (LRFS). The secondary endpoints were locoregional recurrence-free survival (LRRFS), distant metastasis-free survival (DMFS), and overall survival (OS). PFS was calculated from the date of randomization to the date of progression or death. LRFS was calculated from the date of randomization to the date of documented recurrence in the nasopharynx or death from any cause. LRRFS was calculated from the date of randomization to the date of documented locoregional recurrence. DMFS was calculated from the date of randomization to the date of documented distant metastasis, and OS was calculated from the date of randomization to patient death.

We employed the χ2 test to analyze classification variables. Kaplan–Meier survival curves were used to compare time-to-event data between the DWI and MRI groups. Univariate and multivariate survival prognostic analyses were performed using the Cox proportional hazards model. We calculated the sample size by PASS software. All analyses were performed using SPSS (version 27.0), with statistical significance set at *P* value < 0.05.

## Results

### Patient characteristics

Between October 2021 and December 2023, 185 patients diagnosed with recurrent NPC were screened for eligibility, 150 of whom were enrolled for either DWI-guided IMRT (*n* = 75) or conventional MRI-based IMRT (*n* = 75). Table 1 outlines the baseline patient characteristics. No significant differences in clinical features or baseline demographics were observed between groups.

**Table 1:** Baseline clinical characteristics

Characteristics	DWI-Guided DP-IMRT group No. of patients	Conventional MRI-based IMRT No. of patients	*P* value
Total	75	75	
Age, y			
Median	53	55	
Range	18–70	22–70	
Sex			0.198
Male	65	59	
Female	10	16	
Karnofsky Scale			0.119
90–100	67	72	
70–80	8	3	
EBV-DNA			0.072
≥ 400 copies/ml	47	36	
< 400 copies/ml	28	39	
Recurrent stage			0.413
III	45	40	
IV	30	35	
Recurrent T Stage			0.174
rT1	11	6	
rT2	11	13	
rT3	34	29	
rT4	19	27	
Recurrent N Stage			0.886
rN0	33	32	
rN1	10	7	
rN2	20	27	
rN3	12	9	0.056
Median time interval between 1st and 2nd course of RT (years, range)	2.3 (1.6–4.9)	2.4 (1.8–4.5)	

### Treatment compliance and response

In the DWI group, 69 patients (92%) completed ≥ 2 cycles of IC, while 68 patients did so in the MRI group. Suspension in the third cycle of IC occurred because of grade 4 oral mucositis and severe hematological toxicity. Two patients did not receive the full planned dose of radiotherapy because of patient refusal and severe hematological toxicity. The mean duration of radiation therapy was 1.5 months. Six months after the completion of radiotherapy, five patients with residual neck lymph nodes were observed (two patients in the DWI group and three patients in the MRI group), and salvage surgery was successfully performed.

### Adverse events

The adverse events are listed in Table 2. Both groups showed similar toxicities. Twelve patients in the DWI group and 10 patients in the MRI group (grades 3 or 4) experienced acute toxic effects, including anemia, neutropenia, leukopenia, and thrombocytopenia. The most common late toxicities were mucosal necrosis and central nervous system injury. Fortunately, no grade 5 toxicities (death) occurred in either group.

**Table 2:** Grade 3 to 4 toxicity

Adverse events	DWI group	MRI group	*P* value
	No. of patients (%)	No. of patients (%)	
Anemia	2 (2.7)	2 (2.7)	0.758
Neutropenia	4 (5.3)	4 (5.3)	0.905
Leukopenia	5 (6.7)	4 (5.3)	0.527
Thrombocytopenia	1 (1.3)	0	0.165
Liver dysfunction	0	0	-
Nephrotoxicity	0	0	-
Fatigue	0	0	-
Nausea	0	0	-
Vomiting	0	0	-
Oral mucositis	0	0	-
Hyponatremia	0	0	-
Hoarseness	0	0	-
Late adverse events			
Trismus	4 (5.3)	6 (8.0)	0.465
Dysphagia	8 (10.7)	9 (12.0)	0.156
Mucosal necrosis	18 (24.0)	21 (28.0)	0.580
Central nervous system injury	16 (21.3)	14 (18.7)	0.686
Dry mouth	1 (1.3)	2 (2.7)	1.000
Ototoxicity	0	0	-
Massive epistaxis	3 (4.0)	5 (6.7)	0.165

### Treatment failure

The median follow-up time was 16 months (range: 6–27 months). Fourteen (18.7%) patients in the DWI group and 24 (32.0%) in the MRI group had locoregional tumor recurrence. Among these patients, thirteen patients in the DWI group and 22 in the MRI group had in-field recurrence (in which 95% or more of the recurrence volume was within the 95% isodose); One patient in the DWI group and 2 in the MRI group had outside-field recurrence (in which less than 20% of recurrence volume was within the 95% isodose). Five patients in the DWI group and seven in the MRI group experienced distant metastases. Among these patients, two, two, and three had lung, liver, and bone metastases, respectively. Five patients had metastases to more than one organ.

### Survival

**Fig. 3:**
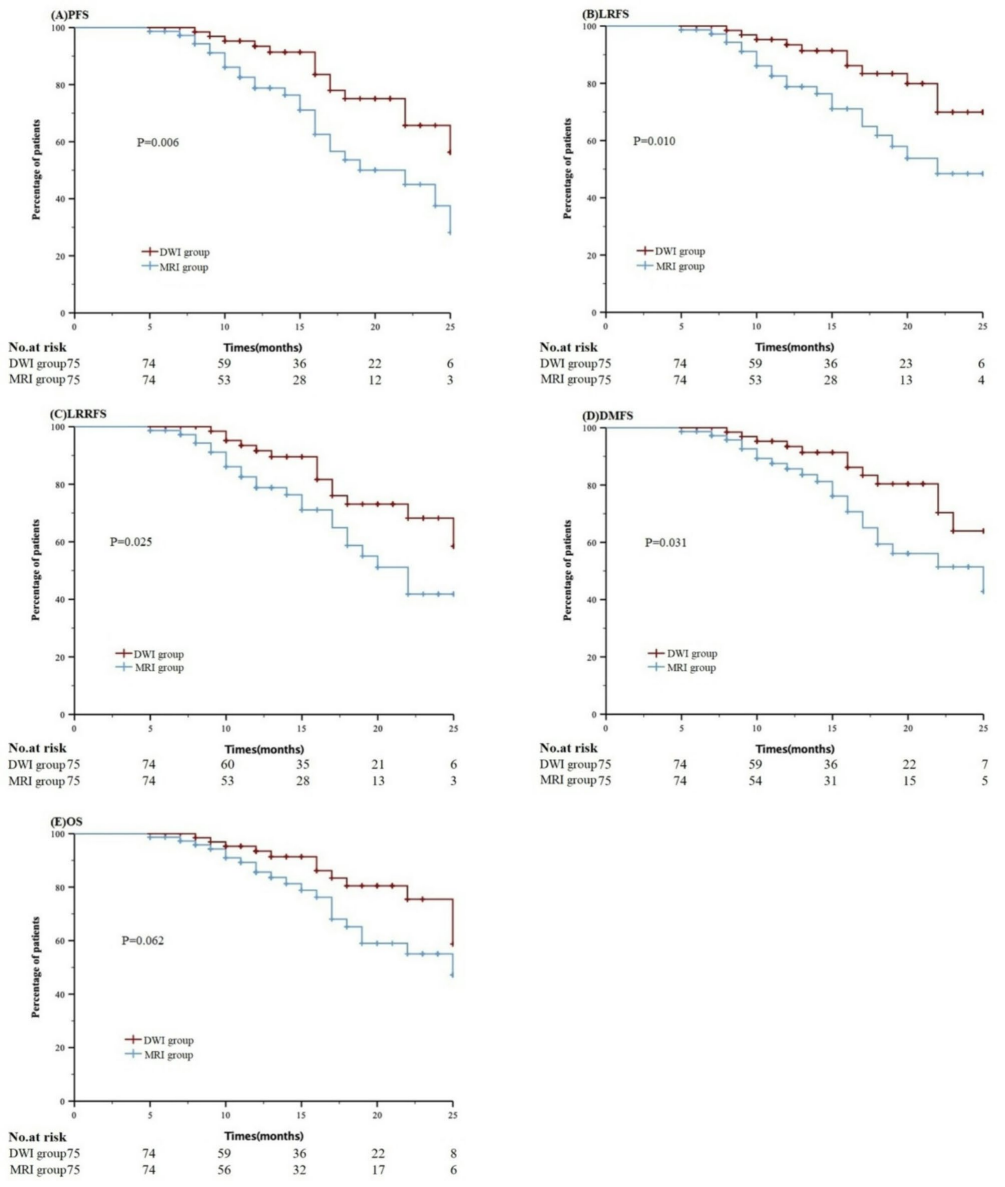
Kaplan-Meier survival curves for DWI-guided DP-IMRT and Conventional MRI-based IMRT without DP groups. PFS (**A**), LRFS (**B**), LRRFS (**C**), DMFS (**D**), OS (**E**)

Thirty-three deaths (12 in the DWI group and 21 in the MRI group) were reported. In the DWI group, the causes of death were local and distant failure (four patients), massive nasal bleeding (six patients), and cachexia (two patients). In the MRI group, death was due to distant metastases (two patients), local failure (six patients), temporal lobe necrosis (three patients), massive nasal bleeding (eight patients), or unknown reasons (two patients). The 18-month PFS rate was 75.1% in the DWI group and 53.6% in the MRI group (*P* = 0.006). The 18-month LRFS rate was 83.4% in the DWI group and 61.8% in the MRI group (*P* = 0.010). The 18-month LRRFS rate was 73.1% in the DWI group and 64.9% in the MRI group (*P* = 0.025). The 18-month DMFS rate was 80.0% in the DWI group and 65.1% in the MRI group (*P* = 0.045). Patients in the DWI group had a higher 18-month PFS, LRFS, and DMFS than those in the MRI group (Fig. 3). Furthermore, the 18-month OS rate was 80.5% in the DWI group and 65.2% in the MRI groups (*P* = 0.062).

### Prognostic factors

The univariate analysis revealed that DWI-guided DP-IMRT was an important prognostic factor for 18-month PFS and LRFS (*P* = 0.041 and *P* = 0.030, respectively). Age was an important prognostic factor for PFS, LRFS, LRRFS, DMFS, and OS. The multivariate analyses further confirmed that DWI-guided DP-IMRT was an independent prognostic indicator of 18-month PFS and LRFS (*P* = 0.012 and *P* = 0.019, respectively). The detailed results of the multivariate analysis are presented in Table 3.

**Table 3:** Multivariable analysis of prognostic factors in locally advanced head and neck squamous cell carcinoma. Age (≥ 53 vs. < 53 years), sex (male vs. female), pretreatment EBV-DNA level (≥ 400 vs. < 400 copies/mL), tumor stage (T1-2 vs. T3-4), KPS (90–100 vs. 70–80), node stage (N0-1 vs. N2-3)

Endpoint	HR (95% CI)	*P* value
Progression-free survival		
Sex	1.177 (0.342–4.055)	0.796
Age	2.129 (1.043–4.347)	0.038
T stage	0.551 (0.201–1.511)	0.247
N stage	1.061 (0.504–2.234)	0.877
Karnofsky scale	1.061 (0.496–6.144)	0.385
EBV-DNA	1.847 (0.919–3.710)	0.085
DWI-guided dose painting	0.427 (0.220–0.828)	0.012
Local recurrence-free survival		
Sex	1.136 (0.326–3.957)	0.842
Age	2.417 (1.087–5.373)	0.030
T stage	0.454 (0.155–1.325)	0.148
N stage	0.736 (0.335–1.617)	0.445
Karnofsky scale	1.975 (0.437–8.918)	0.376
EBV-DNA	1.854 (0.869–3.956)	0.110
DWI-guided dose painting	0.410 (0.195–0.865)	0.019
Locoregional recurrence-free survival		
Sex	0.918 (0.307–2.744)	0.878
Age	2.225 (1.061–4.666)	0.034
T stage	0.389 (0.137–1.105)	0.076
N stage	0.799 (0.385–1.657)	0.547
Karnofsky scale	2.074 (0.465–9.250)	0.339
EBV-DNA	1.493 (0.743–3.003)	0.260
DWI-guided dose painting	0.526 (0.268–1.034)	0.063
Distant metastasis-free survival		
Sex	1.104 (0.318–3.826)	0.877
Age	2.279 (1.030–5.044)	0.042
T stage	0.419 (0.144–1.224)	0.112
N stage	0.816 (0.373–1.783)	0.610
Karnofsky scale	1.813 (0.407–8.086)	0.435
EBV-DNA	1.514 (0.729–3.146)	0.266
DWI-guided dose painting	0.511 (0.249–1.048)	0.067
Overall survival		
Sex	1.067 (0.308–3.697)	0.918
Age	2.288 (1.032–5.070)	0.042
T stage	0.397 (0.136–1.156)	0.090
N stage	0.694 (0.314–1.534)	0.367
Karnofsky scale	1.790 (0.400-8.000)	0.446
EBV-DNA	1.703 (0.807-0.3.593)	0.162
DWI-guided dose painting	0.557 (0.269–1.156)	0.116

Abbreviation: EBV-DNA = Epstein-Barr virus DNA; HR = hazard ratio

## Discussion

Recurrence is a major cause of death in patients with NPC [24]. Reirradiation is the recommended treatment for locally recurrent NPC. The reirradiation dose is one of the most significant factors affecting treatment efficacy. Boosting the radiation dose to the GTV can improve local recurrence; however, it is limited due to the increase in radiation-related toxicities [20, 25]. Heterogeneity is an important source of variation in target volume selection and delineation of tumors. This heterogeneity can be quantified using the ADC, which increases the LC. To our knowledge, the benefits of maximizing locoregional control after reirradiation should be carefully balanced with the risk of radiation-related side effects [26]. DWI-guided IMRT can improve the treatment effect in HNSCC and NPC [12, 27–29].

DWI has a higher functional ADC contrast and has already shown value in treatment response evaluation and tumor detection in NPC and other cancers [13, 29–31]. This enabled the precise design of the GTV, delivery of the optimal dose to the target volume, and dose constraints to the OARs. Our previous study [29] showed that DWI-guided DP-IMRT had a DFS benefit among patients with locally advanced NPC, and toxicity did not show differences in the short-term follow-up. Felice et al. [32] suggested that using ADC-guided GTV delineation in the era of IMRT planning may be a safe way to reduce the uncertainty in delineating tumor volume and, consequently, geographical misses. DWI has been suggested as a predictive factor for tumor response to chemoradiotherapy [33, 34]. Therefore, DWI-guided IMRT is required to improve LC.

In this trial, we chose ADC < mean as the standard clinical delineation of GTV for dose escalation, consistent with previous clinical investigations wherein DP by contours was performed based on regions with ADC < mean [30]. ADC-based targets are the leading cause of dose differences. DWI-guided DP-IMRT, as an effective technique for dose escalation, can significantly increase the biologically effective dose delivered. Therefore, we chose to increase the biologically effective dose by about 10% (79.66 Gy) in the DWI group compared to 72 Gy in the MRI group.

In our study, most patients (98.6%) completed the full planned dose of radiotherapy. The median follow-up duration was 16 months. Our findings based on the DWI-guided DP-IMRT regimen showed benefits compared with those of other studies delivering conventional MRI-based IMRT in patients with NPC [29, 35]. Guan et al. [36] reported that compared with radiotherapy alone, concomitant chemoradiotherapy for patients with locally recurrent NPC increased the 3-year OS rate from 42.2 to 68.7%. In a multicenter, open-label phase 3 trial by Chen et al. [21], 72 patients with stage recurrent II–Iva carcinoma received conventional MRI-based IMRT; the 3-year OS rate was 55.0%.

Previous studies have indicated that DWI-guided dose escalation has significant benefits in patients with locally advanced NPC. Law et al. [37] found that the skewness of the ADC distribution is an independent predictor of local recurrence in patients with NPC. Our previous study showed that DWI-guided DP-IMRT was a prognostic factor for DFS in patients with LA-NPC [29]. The present study shows that DWI-guided DP-IMRT has survival benefits in patients with recurrent NPC.

As reported by Skorska et al. [38] and in our previous study [29], acute toxicities did not increase with the radiation dose of DWI-guided DP-IMRT, and patients showed good tolerance to this treatment compared with conventional MRI-based IMRT. Previous studies showed that approximately 31.3–40.0% of patients who received a 60 Gy reirradiation dose in radiotherapy died from severe radiation-induced complications [22, 39]. Our study confirmed that the toxicity was within the range of reported toxicities for recurrent local NPC treated with reirradiation. In the DWI group, 5.3% of patients developed trismus, 10.7% of patients developed dysphagia, 24% of patients developed mucosal necrosis, 21.3% of patients developed central nervous system injury, and 1.3% of patients developed dry mouth, which is common late radiation toxicity. In the conventional MRI-based IMRT group, 8% of patients developed trismus, 12% of patients developed dysphagia, 28% of patients developed mucosal necrosis, 18.7% of patients developed central nervous system injury, and 2.7% of developed dry mouth. The late toxic effects were similar between treatment groups. These results are consistent with the 33–45% incidence rate of toxicity in recurrent NPC reported in another study [21, 40, 41]. A randomized phase II trial indicated that acute and late toxicities were similar between the two groups receiving standard chemoradiotherapy (70 Gy/35 fx) or dose escalation using DP (77 Gy/35 fx), consistent with the results of our study [42, 43]. Furthermore, in a study by Welz et al. [42], the acute toxicity levels reported in the high-dose DWI and standard IMRT groups did not show significant differences.

### Limitation

This trail had some limitations that should be acknowledged. Firstly, all patients took treatments at Hunan cancer hospital, which might limit the generalizability of the treatment results to non-endemic NPC area. Additionally, further follow-up is needed to assess the long-term survival outcomes of patients with recurrent nasopharyngeal carcinoma.

## Conclusion

Compared with conventional MRI-based IMRT, DWI-guided DP-IMRT had a higher PFS and LRFS benefit, with no different toxicity, among patients with recurrent locally advanced NPC.

## Electronic supplementary material

Below is the link to the electronic supplementary material.

Supplementary Material 1

## Data Availability

The original contributions presented in the study are included in the article. Further inquiries can be directed to the corresponding authors.
